# Maintaining essential health services during COVID-19 in Ghana: a qualitative study

**DOI:** 10.1136/bmjgh-2023-013284

**Published:** 2024-03-15

**Authors:** Isaac Yeboah, Duah Dwomoh, Rawlance Ndejjo, Steven Ndugwa Kabwama, Fidelia Ohemeng, Sylvia Akpene Takyi, Ibrahim Issah, Serwaa Akoto Bawuah, Rhoda Kitti Wanyenze, Julius Fobil

**Affiliations:** 1 Employment and Society, University of Professional Studies, Legon, Ghana; 2 Department of Biostatistics, University of Ghana, Legon, Ghana; 3 Disease Control and Environmental Health, Makerere University College of Health Sciences, Kampala, Uganda; 4 Makerere University, Kampala, Uganda; 5 Department of Sociology, University of Ghana, Legon, Ghana; 6 Department of Biological, Environmental, and Occupational Health, University of Ghana, Legon, Ghana; 7 School of Public Health, Makerere University, Kampala, Uganda

**Keywords:** COVID-19, health systems, qualitative study

## Abstract

**Introduction:**

Evidence suggests that non-pharmaceutical interventions such as lockdown policies, restriction of movement and physical distancing to control the novel COVID-19 contributed to the decline in utilisation of essential health services. We explored healthcare providers’ and policy-makers’ experiences of the barriers, interventions and response actions that contributed to ensuring the continuity of essential health services during the COVID-19 pandemic in Ghana to help inform future practice and policy.

**Methods:**

We used a qualitative study approach. Data were analysed using thematic analysis. Thirty Four participants composed of 20 healthcare providers and 14 policy-makers who worked across regions with low and high recorded COVID-19 cases in Ghana during the COVID-19 pandemic were involved in this study.

**Results:**

Participants reported that essential health services including maternal, reproductive and child health services, communicable and non-communicable disease care, and elective surgeries were disrupted during the COVID-19 pandemic. Barriers to the utilisation of essential services were constructed into three subthemes: (1) fear, (2) poor quality of care at the facility and (3) financial limitation. These barriers were mitigated with population-based interventions underpinned by the socioecological model at the individual and interpersonal level (including psychosocial care for families and home visits), institutional and community levels (such as allocation of funds, training of health workers, public education, triage stations, provision of logistics, appointment scheduling, telemedicine and redeployment of health workers) and public policy level (tax relief packages, transportation arrangements and provision of incentives), which helped in maintaining essential health services during COVID-19.

**Conclusion:**

Disruption of essential health services during COVID-19 in Ghana instigated population-based interventions which aided in expanding the populations’ continuous access to essential health services and strengthened health service delivery.

**WHAT IS ALREADY KNOWN ON THIS TOPIC**

**WHAT THIS STUDY ADDS**

- COVID-19-related disruptions forced health service authorities to initiate strategies that meet patient and health professional needs.
- Population-based interventions were adopted at the intrapersonal, interpersonal, community, institutional and public policy levels.

**HOW THIS STUDY MIGHT AFFECT RESEARCH, PRACTICE OR POLICY**

- Essential healthcare services must be prioritised in planning and responding to future pandemics to minimise healthcare system shocks.
- The government of Ghana through the Ghana Health Service should build resilient health facilities by strengthening health facility readiness and preparedness for pandemic response and also set up emergency funds to respond to future pandemics.
- We recommend mixed-methods evaluations of population-based interventions at various sublevels of the population and health system to inform policy and practice.

## Introduction

 Epidemics and pandemics including Ebola, SARS-CoV-2 and COVID-19 contributed to increased morbidity and mortality.^1 2^ Essential Health Services (EHS) encompass services with high priority, guided by a country’s health system and burden of disease, aimed at preventing communicable diseases, reducing maternal mortality and child morbidity, preventing acute chronic conditions and managing emergency conditions.^3^ EHSs also cover critical treatments, medications, inpatient therapies, lab and blood bank services, disease prevention, reproductive healthcare, and support for vulnerable populations such as infants and adults.

Pandemics have supply and demand impacts that lead to supply declines and reductions in the usage of EHSs. On the supply side, EHSs are disrupted and reduced, in part resulting from unavailability or infection of health workers and shortage of essential drugs.^4^,^7^ Other supply dynamics of decline in the use of EHSs are movement restriction policies including curfews, transport closures and stay-at-home orders.^8^,^11^ On the demand side, clients’ fear of getting infected with infectious diseases results in a decrease in the use of EHSs.^1 2^

As COVID-19 spread, EHSs in sub-Saharan Africa were severely impacted. Limited research has focused on COVID-19’s effects on accessing these services.^12 13^ Recognising barriers and adapting strategies is crucial for future pandemic responses. Studies show diverse COVID-19 health strategies have been employed to maintain EHSs, including telemedicine, home drug delivery, extended working hours.^14^,^16^ While research often focuses on developed nations,^15^,^17^ understanding challenges in developing nations is crucial.^18^ This study aims to comprehend challenges and strategies in developing countries’ health contexts using the socioecological model. The socioecological model proposes population-based interventions at individual and interpersonal levels, institutional and community levels, and public policies to sustain EHSs.^19^ This study seeks to understand how EHSs in Ghana during the COVID-19 pandemic were maintained, identifying the barriers that remained and reflecting on interventions to inform future health system planning and pandemic preparedness.

## Methods

### Design

This descriptive exploratory study had a qualitative design using a semistructured interview guide. The study features interpretative phenomenological analysis and narrative approaches. Front-line health workers shared their lived experiences of how EHS were maintained as required in the phenomenology approach to research.^20^ On the other hand, policy-makers shared experiences and knowledge about how EHS were maintained using the narrative approach.^21^ A thematic analysis was used as a tool to identify key themes emerging from the interviews. A deductive approach was chosen to better focus on the experiences and perspectives of front-line healthcare providers and policy-makers. The design and reporting for this study were guided by the Consolidated Criteria for Reporting Qualitative Research guidelines.^22^

### Settings

The study was conducted in Greater Accra, Ashanti, Oti, Northern and Western regions of Ghana. Greater Accra, Ashanti and Western regions were representative of regions that recorded high cases of COVID-19 while Oti and Northern regions recorded low cases of COVID-19.

### Participants, sampling and recruitment

Individuals were considered eligible if they were front-line healthcare workers, policy-makers or other stakeholders who worked during the COVID-19 pandemic in regions with either low or high recorded COVID-19 cases. Participants were recruited using purposive sampling. We recruited health workers who worked in health facilities during the COVID-19 pandemic, whom we refer to as front-line health workers. Policy-makers and stakeholders who were directly involved with decision-making at the community, district, regional and national levels regarding COVID-19 were also recruited. Recruitment took place between March and May 2021 in all five regions of the study. No participant declined to take part in the study once they agreed to take part. There were no repeat interviews with the same participants. We stopped recruiting study participants due to the richness of data gathered, in-depth understanding of participants’ perspectives and the absence of new ideas emerging. A total of 34 participants (20 front-line health workers and 14 policy-makers) were involved in this study.

### Data collection

The purpose and significance of the study were communicated to study participants and interviews were scheduled at the convenience of the study participants. Male and female interviewers who had just graduated with a Master in Public Health and experience in qualitative interviews were recruited as research assistants to conduct interviews. In addition, IY conducted five of the interviews with policy-makers. Front-line health workers were recruited from secondary, tertiary and quaternary health facilities. Policy-makers and other stakeholders were recruited from the national COVID-19 task force, regional rapid response team, municipal/district rapid response team, Ghana Health Service, Municipal health directorate and district health directorate. Two different interview guides were prepared before conducting interviews; one for front-line health workers and the other for policy-makers and stakeholders. The interview guide for front-line health workers consisted of questions on services disrupted, challenges to utilisation of EHS and facility strategies to provide EHS. The interview guide for policy-makers and stakeholders elicited information on disruptions of EHS, challenges to maintaining/restarting EHS and health system interventions to maintaining or restarting EHS. All the interview guides were in English. In addition, all interviews were conducted in English with most of the interviews at the office of the selected participants. Physical distance was maintained during face-to-face interviews while both the research assistant and research participant wore face masks. All interviews were done on a face-to-face basis. Simultaneously, interviews were conducted across all selected facilities. Following each interview, transcription and coding were conducted to generate and refine themes. Interviews lasted 1 hour to 1 hour and 30 min. After the interview, research assistants provided key points of the interview to validate the data as a form of checking.^23^ The research team did not have any contact with participants before obtaining informed consent. During the interviews, there was no one present besides the participants and researchers. Research assistants wrote field notes daily. Transcripts were shared with authors to review and provide feedback. The iterative nature of this process continued until after the 34th participant, at which point we discerned a comprehensive and refined coding structure, signifying that data saturation had been achieved. In primary qualitative research, the concept of saturation, indicating that no new information or themes are emerging, served as the criterion for concluding data collection. The daily feedback was done until meaningful saturation was reached.^24 25^ Therefore, the decision to conclude data collection was based on achieving a state of saturation, ensuring a thorough exploration of the research questions.

The study was guided by phenomenological approach based on the premise that barriers and strategies to maintaining EHS are best understood from the experiences of front-line health workers, and policy-makers of policies and programmes.

### Data analysis

All interviews were audio-recorded with permission from participants, transcribed verbatim, exported to NVivo (V.10) and analysed using the six steps of thematic analysis described by Braun and Clarke.^26^ The six steps of thematic analysis involve (1) data familiarisation, (2) generating initial codes, (3) identifying preliminary themes, (4) reviewing themes, (5) defining and naming themes and (6) reporting. First, the entire transcripts were read for an overall understanding of the data. Second, codes addressing research questions were identified. Third, the initial codes identified were synthesised into wider themes. The themes from front-line health workers and policy-makers were reviewed and synthesised. In the fifth stage, we drew up figures for thematic analysis where data for each theme were collected from all interview transcripts. The final step involved the preparation of the manuscript with quotes from the interview data to illustrate the themes identified. Two coauthors (IY and DD) monitored the entire process of data analysis.

### Patient and public involvement

Patients and/or the public were not involved in the design, conduct, reporting or dissemination of this research.

## Results

Table 1 presents the demographics of the participants. 20 in-depth interviews were conducted with front-line health workers while 14 key informant interviews were conducted with policy-makers. In addition, a detailed description of the participant is provided in tables2  3.

**Table 1:** Characteristics of participants

Characteristics	Front-line health workers (N=20)n (%)	Policy-makers (N=14)n (%)
Age		
24–34	3 (15.0)	0 (0.0)
35–49	15 (75.0)	7 (50.0)
50–59	1 (5.0)	5 (35.7)
60+	1 (5.0)	2 (14.3)
Marital status		
Married	18 (90.0)	11 (78.6)
Not married	1 (5.0)	3 (21.4)
Separated	1 (5.0)	0 (0.0)
Type of facility		
Secondary	2 (10.0)	
Tertiary	15 (75.0)	
Quaternary	3 (15.0)	
No of living children		
None	2 (10.0)	1 (7.1)
1	1 (5.0)	5 (35.7)
2	12 (60.0)	5 (35.7)
3	3 (15.0)	3 (21.4)
4	2 (10.0)	0 (0.0)
Region		
Greater Accra	6 (30.0)	5 (35.7)
Oti	5 (25.0)	1 (7.1)
Ashanti	5 (25.0)	3 (21.4)
Western	0 (0.0)	4 (28.6)
Northern	4 (20.0)	1 (7.1)
Institution		
District Health Directorate		2 (14.3)
Municipal Health Directorate		6 (42.8)
Ghana Health Service		1 (7.1)
Presidency		1 (7.1)
Medical Research Institute		1 (7.1)
Tertiary facility		1 (7.1)
Municipal assembly		1 (7.1)
District assembly		1 (7.1)
Level of team		
National COVID-19 Task Force		3 (21.4)
National case management team		3 (21.4)
District rapid response team		3 (21.4)
Municipal rapid response team		5 (35.7)

**Table 2:** Detailed profile of policy-makers/stakeholders

Participant ID	Institution	Profession/specialty	Level of team	Region
KII1	Korle-Bu Teaching Hospital	Biomedical scientist	National Case Management Team	Greater Accra
KII2	Noguchi Memorial Institute for Medical Research	Epidemiologist	National Case Management Team	Greater Accra
KII3	District Health Directorate	Public health specialist	District Rapid Response Team	Western Region
KII4	Municipal Assembly	Government appointee	Municipal/Regional Rapid Response Team	Western Region
KII5	Municipal Health Directorate	Public health specialist	Municipal/Regional Rapid Response Team	Western Region
KII6	Municipal Health Directorate	Public health specialist	Municipal/Regional Rapid Response Team	Western Region
KII7	Ghana Health Service—Public Health Division	Medical doctor	National COVID-19 Task Force	Greater Accra
KII8	Presidential Health Advisory	Medical doctor	National COVID-19 Task Force	Greater Accra
KII9	Noguchi Memorial Institute for Medical Research	Virologist	National Case Management Team	Greater Accra
KII10	Metropolitan Health Directorate—Ghana Health Service	Public health specialist	Regional Response Team	Ashanti Region
KII11	Metropolitan Health Directorate—Ghana Health Service	Medical doctor	Regional Response Team	Ashanti Region
KII12	Metropolitan Health Directorate—Ghana Health Service	Public health specialist	Regional Response Team	Ashanti Region
KII13	Municipal Health Directorate	Public health specialist	Municipal/Regional Rapid Response Team	Oti Region
KII14	Municipal Health Directorate	Medical Doctor	National COVID-19 Task Force	Northern Region

**Table 3:** Detailed profile of front-line health workers

Participant ID	Unit/specialty	Level of facility	Region
IDI1	Nurse	Tertiary	Northern Region
IDI2	Head of microbiology department	Tertiary	Greater Accra Region
IDI3	Head of disease control	Tertiary	Ashanti Region
IDI4	IPC consultant	Tertiary	Ashanti Region
IDI5	Nurse	Tertiary	
IDI6	Principal nursing officer	Tertiary	Greater Accra
IDI7	Disease control officer	Tertiary	Greater Accra
IDI8	Head of laboratory	Quaternary	Greater Accra
IDI9	Midwife	Quaternary	Greater Accra
IDI10	Emergency unit	Tertiary	
IDI11	Pharmacist	Quaternary	Greater Accra
IDI12	Medical doctor	Tertiary	Northern Region
IDI13	Nurse manager, accident and emergency department	Tertiary	Northern Region
IDI14	Pharmacist	Secondary	Northern
IDI15	Disease control officer	Secondary	Oti Region
IDI16	Medical laboratory scientist	Private tertiary	Oti Region
IDI17	Nutrition officer	Private tertiary	Oti Region
IDI18	Medical director	Private tertiary	Oti Region
IDI19	Pharmacist	Tertiary	Oti Region
IDI20	IPC dept	Tertiary	Greater Accra

IPC, interpretative phenomenological analysis .

The findings consisted of 3 main themes and 20 subthemes: (1) disruption of essential services, (2) barriers to utilisation of EHS and (3) strategies to maintaining EHS. See online supplemental table S1 for an overview of the themes and subthemes with sample supporting quotes.

### Theme 1: disruption of essential services

This theme outlines EHSs disrupted at the different health facility levels. Front-line health workers and policy-makers highlighted essential services that were interrupted during the pandemic, which may contribute to increasing preventable morbidity and mortality. This is discussed under three subthemes: (1) maternal, reproductive and child health, (2) communicable and non-communicable diseases and (3) elective surgeries.

#### Subtheme 1: maternal, reproductive and child health

Maternal and child health services decreased during the pandemic, affecting women and children across regions with varying COVID-19 cases. Both low and high case regions experienced disruptions in essential services such as antenatal care, obstetrics, gynaecology and routine immunisations, with obstetrics and gynaecology particularly impacted in regions with fewer cases.

Uh, well at that time, I was at the obstetrics and gynecology (ONG). This is where women and babies, and then the big babies attend. It [obstetrics and gynaecology services] was affected. I would think that one was hardly hit. (Frontline Health Worker, Northern Region)

Our services, our immunization, routine activities were disrupted. Besides, caretakers were scared to bring their children for weighing sessions. (Frontline Health Worker, Oti Region)

The perspectives of front-line health workers concur with the perspective of policy-makers in regions with low cases of COVID-19.

We realized that ANC, routine immunization, services provided at the OPD were greatly affected over here (Policymaker, Northern Region)

We were so much affected in this region. All the special clinics basically were not working. Some of the special clinics include ART clinic, NCD clinic, etc. They were not attending these clinics at all (Policymaker, Oti Region)

In high-COVID-19 regions, antenatal services were significantly disrupted, leading to reduced utilisation of maternal and reproductive health services. Initial visits for pregnant women were insufficient, resulting in complications and the need for urgent medical care.

Pregnant women accessing antenatal services were told to get their routine medication. So, the regular visits to the antenatal were not there. So, people could suddenly come with complications because you are not monitoring them. (Frontline Health Worker, Ashanti Region)

It means that people were not coming and antenatal cases went down so if antenatal cases went down, the pregnant women were not coming for antenatal care, the risk of consequence maternal deaths and also infant mortality. (Policymaker, Greater Accra Region)

The disparity in the consequences of EHS disruptions during the COVID-19 pandemic was more pronounced in regions with high COVID-19 cases compared with those with low COVID-19 cases. This was evident in the closure of health facilities whereas regions with fewer cases experienced no shutdowns. Regions with high COVID-19 cases had strained healthcare systems, shortage of medical supplies and increased workloads, prompting health facilities to close or limit services to cope with the pandemic.

COVID-19 led to closure of facilities and affected the provision of maternal and child health. OPD attendance declined but not much. In the Northern region, not a single health facility was closed down because we had enough Doctors that we could rely on if any region experienced a shortage of doctors and other healthcare providers. (Policymaker, Greater Accra Region)

Accra and other regions that recorded high COVID-19 cases were much affected in that some facilities were closed down. This is because these regions had a shortage supply of PPEs and most of the health workers got infected (Policymaker, Greater Accra Region).

Furthermore, those with low COVID-19 cases did not encounter many challenges in the health system and had some healthcare providers available to handle existing demand without significant disruptions.

We had health workers in the facilities to complement the work of the frontline healthcare workers. So whenever, the medical doctor is on duty we make sure we assign more than three nurses to assist the physician (Policymaker, Northern Region).

#### Subtheme 2: communicable and non-communicable diseases

In regions with lower COVID-19 cases, the availability of clinics for communicable diseases was significantly impacted. Notably, individuals with tuberculosis tended to avoid healthcare facilities due to concerns about compromised immunity and some only sought medical attention only when their conditions worsened.

TB has similar symptoms with COVID and there was general fear for people who cough. So, when you are coughing, you are to go to the health facility for treatment. Such people turned to hide their condition until it was severe. (Frontline Health Worker, Oti Region)

From the perspective of healthcare workers, the fear of contracting COVID-19 led some patients to practice self-medication for uncomplicated malaria at home. While this aligns with the Ghana National Malaria Treatment guidelines, the stigma associated with visiting a health facility during the pandemic contributed to this trend.

For malaria, a lot more patients engaged in self-medication and refused to attend the health facilities for fear of either contracting the COVID or for fear of being labelled as COVID-19 positive and sent to the isolation centre. (Frontline Health Worker, Oti Region)

On the other hand, in regions experiencing higher COVID-19 cases, communicable and non-communicable clinics were less affected. Health services least affected include outpatient department visits, diarrhoea, malaria and HIV services. Higher COVID-19 cases might lead to an increase in healthcare resources, including provision of PPEs, extra staffing and funding which could help in maintaining the regular operations of health facilities and prevent significant disruptions to healthcare services.

Least affected I will say the primary health services like ANC, diabetics, general Outpatient Department visits, malaria service and diarrhoea were least affected. (Frontline Health Worker, Greater Accra Region)

HIV clients stayed home for longer periods after receiving quantities of their medication. Disruptions in the healthcare supply chain during COVID-19 might have led healthcare providers to dispense larger quantities of medication to ensure a continuous supply for patients. Consequently, this could reduce the frequency of visits to healthcare facilities for medication refills, encouraging longer periods at home.

For HIV clients, more quantities were given to them so could stay home for longer periods. (Frontline Health Worker Greater Accra Region)

#### Subtheme 3: elective surgeries

In hard-hit regions such as Greater Accra, non-urgent surgeries were postponed to prioritise COVID-19 resources and minimise virus spread among patients and staff. A healthcare provider in Greater Accra recounted:

Elective surgeries and specialized cases require a specialist that was not an emergency were more affected because most clients could defer their visit to the clinics…If it’s an elective case it is either we are postponing or the client is deferring probably because some of these doctors are involved in COVID-19 management or the fear of the client is they don’t want to come to the hospital and contract the COVID so if it something that can be postponed, they do that themselves. Or we call to reschedule. (Frontline Health Worker, Greater Accra Region)

### Theme 2: barriers to utilisation of EHSs

The pandemic forced patients to desist from receiving healthcare from healthcare professionals in health facilities. Most healthcare professionals reported that the decline in patients’ attendance in health facilities can be attributed to fear of contracting COVID-19, poor quality of care and financial limitations.

#### Subtheme 1: fear

Front-line healthcare workers noted patients avoiding facilities due to COVID-19 fears, particularly in high-case regions, driven by perceived infection risk and stigma.

They believed that if you entered any of the hospitals here, you had a high risk of contracting the disease. So, they would prefer to stay away. That is why [healthcare provision] declined, not because the people were not there to give the care. (Frontline Health Worker, Northern Region)

As I said earlier, we are very superstitious people. So, there is the fear of contracting the disease when they attend hospitals. (Frontline Health Worker, Greater Accra Region)

Some of the clients for fear of contacting COVID-19 preferred to stay home than coming around. We made good use of Community Information Centre (CIC) to tell the community that measures are put in place to discourage them contracting COVID. Some came, others too remained home (Policymaker, Oti Region)

#### Subtheme 2: poor quality of care at the facility

Healthcare worker attitudes affect patient visits; stigma deters them, especially in high-case areas like Greater Accra. Poor quality of care is expected among healthcare workers from regions with high COVID-19 cases because they might have experienced an overwhelming healthcare system.

There are times when our health workers stigmatize you. They don’t come near you; they behave rudely towards you. (Frontline Health Worker, Greater Accra Region)

#### Subtheme 3: financial challenges

During the peak of the pandemic, job losses hindered income, limiting access to EHSs, especially in high-case urban areas. A policy-maker also noted that services were disrupted with attendance going down due to the lack of funds on the part of patients.

Some of the patients visited facilities that offer free services because they are not able to pay for services. This could be why they do not utilize some essential health services. (Policymaker, Ashanti Region)

### Theme 3: interventions to maintaining EHSs

Population-based interventions influenced by the socioecological theory helped to maintain the utilisation of EHSs. This is discussed under three themes: (1) interpersonal and community levels, (2) institutional and (3) public policy levels.

### Theme 1: interpersonal and community levels

Interpersonal interventions involve social interactions, while community-level interventions target geographical populations for health improvement. We identified psychological support by families and home-based care at these levels.

#### Subtheme 1: psychosocial care by families

Family calls provided emotional support, reduced isolation, relieved stress and built resilience in front-line healthcare workers.

Oh, they [family] were supportive. They used to call to encourage me to do this for God and Country and that they are solidly behind me. (Frontline Health Worker, Ashanti Region)

I was treated very nicely. My dad, for instance, was very caring; he cared for me throughout the period. My [other] family [members] were always calling me to know how I was handling the COVID-19 situation at work. They always told me that the lives of others depend on me and they know I can do it. They cared. (Frontline Health Worker, Greater Accra Region)

#### Subtheme 2: home-based care

Home-based care, prioritised in high-case regions, minimised COVID-19 spread, addressed transportation issues, preserved healthcare resources and provided personalised services.

We visited the homes of pregnant women to provide them with antenatal care services since they weren’t coming to the facilities for healthcare services (Frontline healthcare worker, Greater Accra)

As part of efforts to provide home-based care services, communities were engaged and educated on the risks, modes of transmission, and protective behaviours against the COVID-19 disease. This approach strengthens community resilience and encourages early intervention.

But you know, when you go to the field, sometimes, some of the clients are hesitant to come forward for health care. But we kept on giving them health education by telling them the risks, the modes of transmission, and what they could do to protect themselves, even though they could still have good access to our services. (Frontline Health Worker, Ashanti Region)

### Theme 2: institutional levels

COVID-19 policies include fund allocation, triage stations, patient appointments, logistics provision, training, telemedicine and staff redeployment.

#### Subtheme 1: allocation of funds

The government allocated funds to tackle the COVID-19. This fund was used to raise awareness through public education to promote adherence to safety guidelines. To prepare for future pandemics, a policy-maker recommended setting up emergency funds to enhance emergency preparedness for future health crises.

With public health activities, most of the funds have been reallocated to do COVID-19, which means you will not get the full funding for your other projects or programs that you have set in place and you won’t achieve your targets. (Policymaker, Greater Accra Region)

The country made funding COVID-19 a priority to the extent that the President promised $ 100 million for COVID-19. There should be an emergency fund to cater for such situations. If that health emergency fund is there, it’s the same thing that we have to address and we have to look at that. (Policymaker, Greater Accra Region)

#### Subtheme 2: triage stations

The triage station was established across health facilities in the country. Thus, triage stations were created in regions with low and high COVID-19 cases. Policy-makers and front-line health workers expressed that facilities instituted a station where patients were screened for COVID-19 before entering the health facilities. Establishing a triage station ensured that individuals requiring immediate attention received prompt care, preventing delays in EHSs.

There was a triaging system put in place where, for anyone who came in, you ensured first of all that the person did not have COVID-19 before you proceeded to do anything else. COVID-19 tests were the first things we did for patients who presented. (IDI11—Quaternary facility, Greater Accra Region)

During the peak stage there were teams that were screening people at the entrance of the facility and inside, too. When they picked a signal, they would draw the attention of the committee. (IDI1—Secondary facility, Northern Region)

We treated everyone who came to the hospital as a potential carrier of the coronavirus. If a person comes and he has signs of COVID-19, we quickly do an antigen test. If it’s negative, we continue treating them for the problem they reported; if it’s positive then we do a PCR test; if he has to be admitted, we admit him in isolation. If he has to go home, we give him all the necessary advice for home management. If somebody has mild symptoms, we advise the person not to go to the hospital until their symptoms go away. (KII6—Municipal Rapid Response Team)

#### Subtheme 3: appointment scheduling with patients

Healthcare facilities implemented appointment systems which mitigated staff shortages and ensured continuous service delivery amidst COVID-19.

The system we used helped us to limit overcrowding here and ensured a reduction in the number of trips patients had to make to the hospital (Policymaker, Ashanti Region)

#### Subtheme 4: provision of logistics

The regions that recorded higher COVID-19 cases received more logistics like masks, goggles, sanitisers and medical supplies. Low-case areas, often with poor transportation, faced disparities in logistics distribution

There were cases where you would have about two or three patients who needed ventilator support but there were not enough. Where I work, there was only one ventilator at the intensive care [unit], and a lot of people needed it at a time. (Frontline health worker, Northern Region)

Logistics shortages risk healthcare worker infections, workforce deficits and disrupt essential services such as vaccinations, maternal care and chronic disease management.

There was a shortage of resources like PPEs, medications, and oxygen tanks. It was happening, and those were barriers. There were also delays in retrieving tests as a result of the pressure on testing facilities. (Frontline Health Worker, Oti Region)

Our facility has done very well, but not enough in terms of logistics. Because sometimes you don’t get what you’re looking for—even basic PPEs like nose masks, you go and they give you just a handful of them. (Frontline Health Workers, Northern Region)

#### Subtheme 5: redistribution and rotation of staff

In order not to be crowded at the health facility and increase the risk of infection, health facilities were running a staff rotation (shift) system. This system allowed for fewer nurses in the facility at a time. This was implemented across regions with low and high cases of COVID-19.

I mean, we reset the minimum that was needed so that they came in batches so that if a cohort of a batch got infected, there would be other people to continue the work. (Policymaker, Western Region)

As much as possible, instead of running three shifts, we decided to run two shifts: 8 am to 5 pm and 5 pm to 8 am. This was to ensure that at any point in time, there was no congestion in the hospitals. (Policymaker, Ashanti Region)

Front-line workers redistributed staff and rotated schedules to ensure continuous coverage and address shortages.

One of them was the redistribution of some of the staff. We got staff from units that had more staff. (Frontline Health Worker, Northern Region)

Previously, we were running shifts so that we wouldn’t be stressed. It was an internal arrangement to ensure that when one person got infected, we were sure another person would be able to work. (Frontline Health Worker, Oti Region)

#### Subtheme 6: training

Infection, prevention and control training was conducted for front-line healthcare workers to safeguard their health and the broader community. This training ensured effective management of COVID-19 cases within the healthcare settings.

At a point in time, you also have to train others quickly to reorient them, orient them and some of them shift their task. For instance, a lot of people were not general infection management staff but then the short training to prepare them to join. (Policymaker, Greater Accra Region)

The training, I think every month they were having the training and then most of them they will not tell us, they just inform us, they brought the schedule and so per their schedule we monitor. (Policymaker, Western Region)

#### Subtheme 7: telemedicine

High-COVID-19 regions used telemedicine for remote consultations, triage, preventive care and ongoing health management, ensuring essential service continuity.

Yes, I would say telehealth, because we used a lot of telehealth, especially when we had the waves. People in Accra were [e-meeting] with people who were in Bolga, Tamale, and other places. We used a lot of video conference and health education [tools]. (KII2–National COVID-19 Taskforce)

They brought in some mobile health devices, and so there was mobile health. I remember one called Omni. (KII10–National Case Management Team)

### Theme 3: public policy levels

Public policies for COVID-19 include tax relief, transportation support and incentives.

#### Subtheme 1: tax relief package

The government of Ghana gave tax relief packages to health workers who were in direct contact with the pandemic. This served as a source of motivation for healthcare workers to prioritise their health without facing financial challenges.

During the heat of the crisis to motivate our health workers, government agreed to wave 9 months of their personal tax that they pay or their personal monuments. (Policymaker, Greater Accra Region)

#### Subtheme 2: transportation support

Front-line healthcare workers were supported with the provision of buses that took them to their workplace. This was a policy targeted at healthcare workers working in health facilities with a pressing need for healthcare professionals.

Transport was also provided to take staff in and out of work. (Frontline Health Worker, Oti Region)

Initially, we also transported those who were coming from outside the hospitals and back to their homes during the lockdown so that they didn’t have any problem at all in their minds. To me, that is one of the areas of motivation. (Policymaker, Greater Accra Region)

#### Subtheme 3: incentives

The government gave allowances to front-line workers, up to 50% of their basic salary to serve as motivation for their risky work. This was mainly applied in regions with high COVID-19 cases. Nevertheless, this claim of 50% allowance of front-line workers’ basic salary was disputed as untrue by a health worker in one of the regions with the least cases of COVID-19.

The government gave some allowances as much as 50% of their basic salary as allowances for those who were actually at the frontline. So, it was kind of a motivation. (Policymaker, Greater Accra Region)

I must confess; they took our names, bank accounts, and all those things [with the promise] that they were going to compensate us for our work. After that, not even a penny, I'm telling you. So, as for support, we never had any support. (Frontline Health Worker, Northern Region)

## Discussion

Data from our study highlight that reproductive, maternal and child services were severely affected. With the rapid spread of COVID-19, mothers avoided taking their children to health facilities to seek child health services including immunisation and vaccinations. Similar to our findings, hospitals in urban Uganda saw a decline in antenatal attendance and immunisation services^27^ as hospitals in the Democratic Republic of Congo, Ethiopia, Haiti and Mexico saw a drop in attendance for HIV services, outpatient visits, tuberculosis, malaria, diabetes and hypertension health services.^12 28^ Likewise, a survey of health facilities in India observed that maternal and child health outpatient services were disrupted due to COVID-19.^29^ Patients were deterred from seeking healthcare due to the fear of being stigmatised with COVID-19 as reported in other LMICs.^30 31^ This aligns with the study by Al-Zaman^32^ in Bangladesh that fear and anxiety about the pandemic among communities affected the utilisation of EHSs.

COVID-19 led to deferred surgeries, impacting healthcare provision and potentially increasing morbidity and mortality. Similar findings in Sierra Leone^33^ and Flemish and USA^34 35^ suggest this measure may exacerbate reduced healthcare provision. Despite disruptions and barriers to the utilisation of EHSs, the pandemic spurred population-based interpersonal interventions to enhance health service access and resilience. Positive attitudes were promoted through education and routine home visits. Earlier studies indicate that involving religious leaders in education improved service use in sub-Saharan Africa.^1936^,^38^ Amu *et al*^19^ emphasised educating male partners to support female access to healthcare.

The reassignment of health workers to other units inadvertently led to a reduction in the utilisation of health services such as child health services as found in other studies.^39^ Patients were also given an appointment to reduce pressure for health services. This may put them at risk of not being monitored for preventable health risks and consequently lead to aggravated health conditions of patients. To remedy this situation, efforts should be made by governments of SSA to train more health professionals who provide maternal, reproductive and child health, and elective surgeries. With the upsurge of COVID-19, building a resilient health system in SSA was a challenge. The screening and creation of triage of suspected COVID-19 patients was another intervention implemented to reassure patients of their safety in the health facilities when they visited. Telemedicine, such as the Covid Connect app, facilitated patient–healthcare worker contact, hotspot identification, event attendance, government updates and symptom monitoring during COVID-19 measures. Other studies indicate that telemedicine increases trust and fosters feelings of intimacy and relief when health workers and patients already know each other.^40^,^42^ Studies have demonstrated that the implementation and usage of various telemedicine applications is slow.^43^,^45^ Expanding this variant of telemedicine to cover the provision of EHSs during the COVID-19 pandemic and health services, in general, will strengthen the pursuit of achieving Sustainable Development Goal 3 (SDG).^46^ The government of Ghana in collaboration with other health agencies can work together to initiate mHealth services across the regions to proactively serve the lower-level healthcare facilities. As a form of public policy, the government implemented incentives to front-line health workers and free transportation.^47 48^ This assured the public of availability and readiness to serve their health needs.

### Strengths and limitations of the study

One of the strengths of our study is the triangulation of data collected from front-line health workers, and policy-makers offering insights into disruptions and strategies. Nevertheless, excluding patient perspectives may have limited data comprehensiveness. Future research should include patients’ perspectives.

## Conclusion

This study provides evidence of the impact of COVID-19 on the provision of EHSs. Following conclusions from the study, barriers to the utilisation of EHSs include fear, poor quality of care at the health facility and financial limitations. To tackle the disruption of EHSs, interpersonal and community interventions, institutional interventions and public policy-level intervention levels were employed to maintain EHSs. While achieving SDG 3 is difficult given COVID-19’s disruption of EHSs, there is the need to invest in research, especially in LMICs, to bridge research-practice gaps and make progress towards SDG 3.

## Supplementary material

10.1136/bmjgh-2023-013284 online supplemental file 1

## Data Availability

Data are available on reasonable request.
