# Supplementary material for: Maintaining essential health services during COVID-19 in Ghana: a qualitative study
Source: BMJ Glob Health. 2024 Mar 15;8(Suppl 6):e013284. doi: 10.1136/bmjgh-2023-013284 (PMC11148662; doi:10.1136/bmjgh-2023-013284)
Supplement: online supplemental file 1 [file bmjgh-2023-013284supp001.pdf]

Maintaining Essential Health Services During COVID-19 in Ghana: A Qualitative Study

Supplementary Table S1: Themes identified through interviews with participants

**Global Theme**	**Theme**	**Subtheme**	**Sample Quote**
	1. Disruption of essential services	i. Maternal, Reproductive and Child health	*"Uh, well at that time, I was at the obstetrics and gynecology (ONG). This is where women and baby, and then the big babies attend. It was actually affected. I would think that one was hardly hit"* (IDI1).<br><br>*"I remember in other regions, there were instructions that they should halt immunization services."* (KII1)
		ii. Communicable and Non-communicable diseases	*"TB has similar symptoms with Covid and there was general fear for people who cough. So, when you are coughing, you are to go to health facility for treatment. Such people turned to hide their condition until it is severe."* (IDI1)
		iii. Elective Surgeries	*"If it's an elective case its either we are postponing or the client is deferring probability because some of these doctors are involved in COVID management or the fear of the client is they don't want to come to the hospital and contract the COVID so if it something that can be postponed they do that themselves. Or we call to reschedule.*" (IDI8)
	2. Barriers to utilization of Essential Health Services	i. Fear	*"They believed that if you entered any of the hospitals here, you had a high risk of contracting the disease. So they would prefer to stay away. That is why [healthcare provision] declined, not because the people were not there to give the care."* (IDI12)
		iii. Poor quality of care at the facility	*"There are times when our own health workers stigmatize you. They don't come near you; they behave rudely towards you."* (IDI7)

		vi. Financial challenge	*"They go to the hospitals where testing is free and they're told that their sample collection kits have run out. Then they're forced to go to a place like Noguchi, where they have to pay. That is where some of the patients had problems.*" (KII11)
3. Interventions to maintaining essential health services	i. Intrapersonal and interpersonal levels	*Psychosocial care by families*	
		Home Visits	*"Within the period, antenatal services were not affected much because we used to go to pregnant women in their homes."* (KII1)
	ii. Institutional and Community levels	*Allocation of funds*	*"The country made funding of COVID-19 a priority to the extent that the President promised $100million for COVID-19. There should be emergency fund to cater for such situations. If that health emergency fund is there, it's the same thing that we have to address and we have to look at that."* (KII12)
		Triage Station	*"But we quickly also got around that by introducing measures in terms of triaging, screening patients."* (KII11)
		Appointments scheduling with patients	*"The appointment system helped. It reduced patients' wait time to see a doctor. COVID-19 taught us to use such a system and reduce the number of trips chronic patients had to make to the hospital for a refill of their medication. Our capacity in terms of testing for COVID-19 was lacking, but the appointment system helped to regulate visits to testing sites."* (KII11)

		Provision of Logistics	*"And then every single person in the hospital, and of course, those going out had to be in the right PPE, i.e., face mask, and where possible, goggles."* (KII11)
		Redistribution and rotation of staff	*"I mean, we reset the minimum that was needed so that they came in batches, so that if a cohort of a batch got infected, there would be other people to continue the work."* (KII5)
		Training	*"Staff commitment to training and their readiness to stick to infection prevention control measures really helped us" (KII6)*
		Telemedicine	*"We had an app, COVID Connect, by which people we treated and discharged could keep in contact with us, and people with symptoms could go into the app and indicate their symptoms for some healthcare professionals from our facility to contact them."* (IDI19)
	Public policy levels	*Tax relief package*	*"During the heat of the crisis to motivate our health workers, government agreed to wave 9 months of their personal tax that they pay or their personal monuments."* (KI11)
		Transportation Support	*"Initially, we also transport those who were coming from outside the hospitals and back to the homes during the lockdown so that they didn't have any problem at all in their minds. To me that is one of the areas of motivation." (KII2)*
		Incentives	*"The government gave some allowances as much as 50% of their basic salary as allowances for those who were actually at the frontline. So, it was kind of a motivation."* (KII9)
